# Distinct cross talk of IL‐17 & TGF‐β with the immature CD11c^+^TRAF6^(−/−)^‐null myeloid dendritic cell‐derived osteoclast precursor (mDDOCp) may engage signaling toward an alternative pathway of osteoclastogenesis for arthritic bone loss in vivo

**DOI:** 10.1002/iid3.1173

**Published:** 2024-02-08

**Authors:** Yen Chun G. Liu, Andy Yen‐Tung Teng

**Affiliations:** ^1^ Department of Oral Hygiene Center for Osteo‐immunology & Biotechnology Research (COBR), College of Dental Medicine, Kaohsiung Medical University Kaohsiung Taiwan; ^2^ School of Oral Hygiene & Nursing, and School of Dentistry Kanagawa Dental University (KDU) Yokosuka Kanagawa Japan; ^3^ The Eastman Institute for Oral Health (EIOH), School of Medicine & Dentistry, University of Rochester Rochester New York USA; ^4^ Center for Osteo‐immunology & Biotechnology Research (COBR), School of Dentistry, College of Dental Medicine, Kaohsiung Medical University (KMU) and KMU‐Hospital Kaohsiung Taiwan

**Keywords:** alternative pathway of osteoclastogenesis, arthritic bone loss, IL‐17 & TGF‐β, osteoclast versus CD11c^+^‐myeloid dendritic cell derived osteoclast precursor (mDDOCp), osteo‐immune interactions

## Abstract

**Background:**

Dendritic cells (DCs), though borne heterogeneous, are the most potent antigen‐presenting cells, whose critical functions include triggering antigen‐specific naïve T‐cell responses and fine‐tuning the innate versus adaptive immunity at the osteo‐immune and/or mucosal mesenchyme interface. We previously reported that immature myeloid‐CD11c^+^DCs/mDCs may act like osteoclast (OC) precursors (OCp/mDDOCp) capable of developing into functional OCs via an alternative pathway of inflammation‐induced osteoclastogenesis; however, what are their contribution and signaling interactions with key osteotropic cytokines (i.e., interleukin‐17 [IL‐17] and transforming growth factor‐β [TGF‐β]) to bearing such inflammatory bone loss in vivo remain unclear to date.

**Methods:**

Herein, we employed mature adult bone marrow‐reconstituted C57BL/6 TRAF6^(−/−)^‐null chimeras without the classical monocyte/macrophage (Mo/Mϕ)‐derived OCs to address their potential contribution to OCp/mDDOCp‐mediated osteoclastogenesis in the chicken type‐II‐collagen (CC‐II)‐induced joint inflammation versus arthritic bone loss and parallel associations with the double‐positive CD11c^+^TRAP^+^TRAF6‐null^(−/−)^ DC‐like OCs detected in vivo via the quantitative dual‐immunohistochemistry and digital histomorphometry for analyses.

**Results:**

The resulting findings revealed the unrecognized novel insight that (i) immature myeloid‐CD11c^+^TRAF6^(−/−)^ TRAP^+^DC‐like OCs were involved, co‐localized, and strongly associated with joint inflammation and bone loss, independent of the Mo/Mϕ‐derived classical OCs, in CC‐II‐immunized TRAF6^(−/−)^‐null chimeras, and (ii) the osteotropic IL‐17 may engage distinct crosstalk with CD11c^+^mDCs/mDDOCp before developing the CD11c^+^TRAP^+^TRAF6^(−/−)^OCs via a TGF‐β‐dependent interaction toward inflammation‐induced arthritic bone loss in vivo.

**Conclusion:**

These results confirm and substantiate the validity of TRAF6^(−/−)^‐null chimeras to address the significance of immature mCD11c^+^TRAP^+^DC‐like OCs/mDDOCp subset for an alternative pathway of arthritic bone loss in vivo. Such CD11c^+^mDCs/mDDOCp‐associated osteoclastogenesis through the step‐wise twist‐in‐turns osteo‐immune cross talks are thereby theme highlighted to depict a summative re‐visitation proposed.

## Graphical abstract

The classsical way and understanding for bone resoprtion and loss is mediated by the monocytes/macrophage‐derived osteoclasts. The present study reveals a different alternative pathway for bone resorption and loss, which is done via the immature dendritic cell‐derived osteoclasts with different phenotypic and factional activities: for example, through the step‐wise cytokine cross talks, in an animal model of arthritic bone loss, in vivo

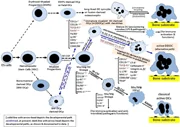

## 1 INTRODUCTION

Bone and bone matrices contain the dynamic skeleton and integral stromal and marrow components with heterogeneous cell types of multilineages, including the immune cells. Dendritic cells (DCs), the potent antigen‐presenting cells, are responsible for activating naïve antigen‐specific T cells, triggering and fine‐tuning the innate versus adaptive immunity and critical interactions to downstream sequelae at the osteo‐immune interface or mucosal mesenchyme.^1^, ^2^, ^3^, ^4^ Recent evidence has suggested that immature DCs of myeloid lineage (mDCs) may likely be involved in inflammation‐induced osteoclastogenesis and bone remodeling, by acting like osteoclast (OC) precursors (OCps) that further develop into DC‐derived OCs (mDDOCp) under inflammatory conditions.^5^, ^6^, ^7^, ^8^, ^9^ DCs can engage T cells and form aggregates^10^, ^11^, ^12^ through RANKL‐RANK signaling under inflammation in vivo,^11^, ^13^ suggesting their engagements with inflammatory bone loss.^1^, ^5^, ^7^, ^9^, ^11^, ^12^, ^13^ Consequentially, these interactions may have further implications, providing potential therapeutic approaches not only for modulating inflammation but also for osteoclastogenic pathways onto bone loss and remodelling.^13^, ^14^

OCs in active functions are multinucleated (≥2–3 nuclei)^7^, ^9^ giant cells, expressing tartrate resistant acid phosphatase (TRAP), calcitonin receptor (CT‐R), cathepsin‐K, and intergrins‐α_v_β_3_ phenotype capable of dissolving the bone matrix.^14^, ^15^, ^16^ OCs are evidently known to be derived from the monocyte/macrophage (Mo/MQ) lineage in the presence of macrophage colony‐stimulating factor (M‐CSF) and receptor activator of nuclear factor kappa B (NF‐κB) ligand (RANKL), which is expressed by multiple cell types, including stromal cells, osteoblasts, chondrocytes, mesenchymal cells and activated T cells, etc.^17^, ^18^, ^19^ RANKL transduces its effects by signaling via its receptor, RANK, for OC differentiation, activity, and survival,^20^, ^21^, ^22^ where its natural decoy/antagonist is osteopotegerin/OPG produced mainly by the stromal and activated immune cells.^14^, ^17^, ^18^, ^19^, ^23^ It is clear that RANKL/RANK‐OPG triad, which is the key adaptor of RANKL/RANK signaling, termed tumor necrosis factor receptor‐associated factor‐6 (TRAF6), is principally associated with transducing pivotal immune/osteotropic interactions through intermediate pathways before capable of triggering transcriptional factors for gene activations,^20^, ^21^, ^22^, ^23^ whose expressions such as NFATc1, DC‐STAMP (for OCp fusion), TRAP, cathepsin‐K, and downstream transcription factors (i.e., PU.1, cFos, MITF, etc.) are critically involved in OC development and osteoclastogenesis,^24^, ^25^, ^26^ thus orchestrating the osteo‐immune cross talks versus immunity as well.^15^, ^19^, ^20^, ^21^, ^22^, ^23^ More so, TRAF6 signaling regulates not only OCs pathophysiology but also wide ranges of immune versus nonimmune activities, differentiation of myeloid cells (DCs & Mo‐Mϕ), and the inflammation‐mediated osteoclastogenesis.^14^, ^15^, ^16^, ^18^, ^19^, ^20^, ^21^, ^22^, ^23^, ^27^, ^28^, ^29^, ^30^

Studies have suggested that DCs, Mo/Mϕ, and OCs could share the common progenitors.^31^, ^32^, ^33^, ^34^, ^35^, ^36^, ^37^, ^38^ As inflammation in the microenvironment may polarize Mo development to either DC or Mϕ^38^, ^39^, ^40^, ^41^, ^42^, tumor necrosis factor‐α (TNF‐α) can skew Mϕ development to DCs from Mo in local tissues, likely through promoting M‐CSF/R upregulation and expression in the circulating OCp pools, suggesting that the proinflammatory TNF‐α signaling may be dependent on the developmental cues in stage‐specific effectors among precursors.^31^, ^41^, ^43^, ^44^, ^45^ Rivollier et al. showed that human Mo‐derived and murine BM‐derived Flt3^+^DCs can transdifferentiate into functional OCs in response to M‐CSF and RANKL in vitro and ex vivo, suggesting that DCs may indeed act like OCps.^3^, ^6^ Further, these myeloid DC‐derived OCs, but not mature or plasmacytoid DCs, have been described, featuring unique environmental and phenotypic characters under immune interactions.^3^, ^6^ However, such developmental cues and the underpinning signal interactions versus molecular mechanism(s) involved in bone loss are largely unclear at present, including their direct contributions per se to inflammatory bone loss for osteoclastogenesis in vivo.^5^, ^6^, ^7^, ^31^, ^32^, ^35^, ^36^, ^37^, ^41^, ^42^, ^46^, ^47^, ^48^

Our lab prior reported that murine bone marrow (BM)/spleen‐derived myeloid DC precursors were able to develop into functional OCs, bearing the immature phenotype (e.g., CD11c^+^CD11b^−^F4/80^−^Ly6C^−^CD31^−^MHC‐II^–/or/low^CD80/86^−^; termed mDDOCp) in vitro and in vivo, capable of resorbing bone in RANKL/RANK‐dependent signaling for differentiation with distinct kinetics and featured morphology of dendrites notable in local environments.^7^, ^9^, ^41^, ^42^ In addition, via the NOD/SCID‐calvarias model, we provided the first amble evidence that mDDOCp/DDOC can indeed develop into CD11c^+^CD11b^−^DC‐like multinucleated OCs under inflammatory conditions for bone loss, supporting its in vivo relevance.^7^, ^9^, ^41^, ^42^ Later, through a genome‐wide microarray screening of CD11c^+^mDCs and parallel neutralization assays in vitro and in vivo, we confirmed that endogenous TGF‐β/TGF‐βRII signaling was essentially involved in developing mDCs/mDDOCp, as OCps, once step‐wise going beyond M‐CSF/R‐mediated differentiation.^7^, ^9^, ^42^ Therefore, we proposed an alternative pathway of inflammation‐induced osteoclastogenesis involving mDCs/mDDOCp, as OCps, and highlighted its potential implications, including their developmental plasticity and regulations as to whether specific mDCs precursor/subsets may serve as the targets for new strategies toward modulating inflammation versus immunity and subsequent bone loss and remodeling.^9^, ^42^

Recently, we investigated the role of TGF‐β in myeloid‐CD11c^+^DCs/mDCs “lacking” TRAF6‐mediated signaling, using total BM cells prepared from syngeneic adult TRAF6^(−/−)^‐toothless mice^20^, ^21^ postlethal irradiation and BM reconstitution [termed: T6KO_bmChi], before being subjected to co‐cultures with and without the naïve CD4^+^T cells and mRANKL or microbial‐*Ag* stimuli in vitro.^7^, ^9^, ^28^, ^30^, ^41^, ^42^, ^49^ Such analyses revealed that TGF‐β signaling was critically involved in developing mDCs/mDDOCp (OCp), where RANKL/RANK‐TRAF6‐mediated signaling were dispensable physiologically and that TRAF6^(−/−)^CD11c^+^mDDOCp became CD11c^+^TRAP^(+)^DC‐like OCs postactivation, indicative of the TRAF6‐independent osteoclastogenesis.^20^, ^21^, ^49^, ^50^ Thus, the adult TRAF6^(−/−)^‐null chimeras may offer an unique approach to assess the effector contributions of OCp/mDDOCp not only in vitro but also its potential use for animal models in vivo as well.^49^, ^50^

To further evaluate and assess the direct contribution and cross‐interactions between mDCs/mDDOCp, as OCp, with the osteotropic cytokine IL‐17 in the absence of Mo/Mϕ‐derived classical OCs to inflammatory bone loss, which remains unclear to date; we applied the established protocols to generate syngeneic mature adult T6KO_bmChi chimeras^50^ and herein to examine the direct contributions of myeloid‐CD11c^+^TRAF6^(−/−)^DC‐like OCs as potential effector in vivo, deficient of the Mo/Mϕ‐derived classical OCs, via chicken type‐II collagen (CC‐II)‐induced joint tissue swelling of the arthritic hind limbs/joints and eroded bone surfaces in the C57BL/6 mice (H‐2^b^), mimicking the (auto)‐inflammatory arthritic bone loss and associations with double‐positive CD11c^+^TRAP^+^TRAF6^(−/−)^DC‐like OCs in the integral tissue sections in situ, properly oriented for detection and measuring the hind limbs’/joints’ bone loss, by the quantitative dual immunohistochemistry (IHC) and digital histomorphometry. Herein, the results of the present study clearly demonstrated that (i) TGF‐β was essentially required to prime immature mDCs/mDDOCp, totally deficient of TRAF6‐mediated signaling, for inflammation‐induced bone loss in vivo as well, via an alternative pathway of osteoclastogenesis despite lacking the Mo/Mϕ‐derived classical OCs, and (ii) IL‐17 engaged the unique interplay during a stepwise signaling seen in (i) above, in the presence or absence of TGF‐β, to mediate a distinct cross talk with CD11c^+^mDCs/mDDOCp before developing the committed TRAF6^(−/−)^CD11c^+^TRAP^+^DC‐like OCs for inflammation‐induced arthritic bone loss in local environment of the susceptible hosts.

## 2 MATERIALS AND METHODS

### 2.1 The TRAF6^(−/−)^‐null chimeric mice and in vivo experiments

**Figure 1:**
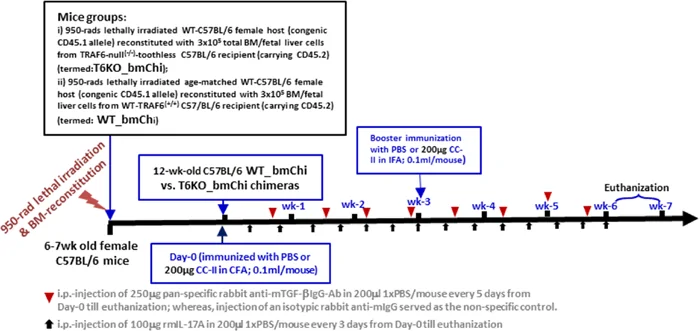
The flow chart of the study protocols employed.

Wild‐type (WT) 4–6‐week‐old female and male C56BL/6 mice were purchased and shipped from the National Laboratory Animal Center, Taiwan, after which they were housed under specific pathogen‐free (SPF; not germ free) conditions with 12‐h controlled climate in the Animal Facilities of Kaohsiung Medical University (KMU). All animal procedures were approved by the local ethics and animal experimentation committees of the Institutional Animal Care and Use Committee of KMU (IACUC protocols #98017 & #98183), Kaohsiung, Taiwan. In addition, syngeneic TRAF6^(+/−)^ heterozygote‐breeding pairs were received as generous gifts from Prof. Y. Choi, Perelman School of Medicine, Univ. of Pennsylvania, Philadelphia, PA, USA, and were placed in the same SPF and controlled‐climate housing on‐site. In the present study, the generation of adult mature TRAF6^(−/−)^‐null mice without the endogenous Mo/Mϕ‐derived classical OCs (termed: **T6KO_bmChi mice**) and the control (termed: **WT_bmChi**) mice reconstituted with the same 3 × 10^5^ total BM/fetal liver cells from the age‐matched WT‐C57BL/6 TRAF6^(+/+)^mice which have been described^49^, ^50^ and thus employed accordingly. Since both syngeneic “donor” WT‐C57BL/6 and TRAF6^(−/−)^KO mice expressed CD45.2‐allele and the co‐isogenic “recipient”‐normal C57BL/6 mice expressed congenic CD45.1‐allele, it allowed evaluations of the donor/CD45.2‐allele versus the recipient‐host/congenic‐CD45.1 BM‐derived cells postirradiation and reconstitution via FACS/flow cytometry analyses for the levels of chimerism produced (in mean ± *SD* % ratio^50^; see Figure 1 flow chart). Notably, both C57BL‐6 recipient WT_bmChi and T6KO_bmChi chimeras survived (>90%–95% survival rate), as housed in our SPF environment at KMU, were all free from any autoimmune or poly‐inflammatory diseases over time (i.e., colitis, etc.^20^, ^21^).

To assess the induction of swelling in arthritic hind limbs/joints for tissue inflammation with bone loss/erosion in C57BL/6 mice (i.e., typically inflamed hind limbs detected in focal areas of the distal tibia or tibiotalar/calcaneum), chicken type‐II collagen (CC‐II) were employed, according to the modified protocols previously reported by Inglis et al.,^51^, ^52^ where 200 μg CC‐II (Chondrex Inc.) in 0.1 M acetic acid was emulsified in the CFA (Sigma‐Aldrich) containing 2.5 µg/µL inactivated *M. tuberculosis* and immunized intradermally using #27‐gauge Hamilton/1 mL syringe at the rump‐shaved two‐sided tail‐base of age‐matched anesthetized WT_bmChi and T6KO_bmChi chimeras (0.1 mL/mouse) under sedation, as marked day 0, followed by a booster injection with the IFA (Sigma‐Aldrich) on day 21. In parallel, age‐matched mice received control phosphate‐buffered saline (PBS) (pH 7.2) in CFA at both days 0 and 21, accordingly (see the schedule in Figure 1 flow chart). Later, for the quantitative immune staining with IHC and digital histomorphometric measures for clinical severity of the hind limbs’/joints’ inflammation and bone loss/erosion, the euthanized mice were then killed on week 6 postimmunization (arthritic/joint inflammation: >50%–60% incidence^51^, ^52^), where the hind limbs/joint samples were harvested for integral tissue sections and prepared as described below for the histology and immune staining with quantitative measures and analyses for bone loss/erosion in situ, as we reported previously.^7^, ^9^, ^28^, ^30^, ^41^, ^42^, ^49^, ^50^, ^53^ To measure the levels of CC‐II‐induced clinical inflammation in vivo, the tissue swelling in hind limbs was evaluated using an Absolute Digimatic Caliper (Model‐999 CDKM, Mitutoyo, Japan; the accuracy/resolution down to 0.01 mm level) from the day of postbooster injection on day 21/week 3 once every 3 days till day 42/week 6 of euthanization.

Subsequently, to address and examine the cytokine's influence on the levels of hind limbs’/joints’ inflammation and bone loss/erosion in vivo, both WT_bmChi and T6KO_bmChi mice were ip injected with 250 μg pan‐specific rabbit anti‐mTGF‐βIgG‐Ab (R&D Systems) in 200 μL 1x PBS on day 0 (*n* = 5x mice/group) and every 5 days thereafter till day 42/week 6 of euthanization^50^; in parallel, mice receiving ip injection with isotypic rabbit anti‐mIgG Ab/serum served as the nonspecific control. Further, to examine the direct influence of exogenous IL‐17 administration on the levels of bone loss/erosion over inflamed hind limbs’/joints’ in vivo, 100 μg/mL rmIL‐17A (R&D Systems) in 200 μL 1x PBS was injected into the mice on day 0 (*n* = ≥5 mice/group/experiment) and every 3 days till day 42 of euthanization, accordingly (see the Figure 1 flow chart).

### 2.2 Histology, quantitative IHC, and histomorphometrical analyses

The histology, dual immunostaining by IHC, and quantitative histomorphometry together enable proper analyses of the target cell types detected for their physical orientations with bona fide tissue locations in situ and associated bone loss/erosion in parallel accordingly, favorably superior to that of the targeted subset(s) analysed by the FACS sorting prepared in the cellular suspensions ex vivo.^7^, ^9^, ^28^, ^30^, ^42^ Thus, to better assess and evaluate the morphological features of multinucleated OCs‐like (≥2–3 nuclei) cells within the tissues collected “in vivo” with/without adjacently colocalized bony changes digitally quantified, the mouse joint samples harvested were timely fixed in 10% formalin, decalcified in Cal‐EX (Fisher Scientific), and paraffin embedded to prepare 4–6‐μm‐thick tissue sections for histology (e.g., hematoxylin and eosin staining) and dual IHC.^7^, ^9^, ^28^, ^30^, ^42^ Immunostained dual IHC was performed using 1 mM ethylenediaminetetraacetic acid at a pH of 8.00 for heat‐induced *Ag*‐retrieval protocol.^7^, ^9^, ^28^, ^30^ Biotinylated hamster anti‐mCD11c‐Ab, Vectastain ABC kit, and Vector NovaRed peroxidase substrate kit (Vectors Laboratories) were used to stain for CD11c (visible in brown), and anti‐TRAP/IgG2b, alkaline phosphatase‐conjugated goat anti‐mouse/IgG2b (Southern Biotech), and Vector‐Blue alkaline phosphatase substrate kit (Vectors Laboratories) were used to staining for TRAP reactivity (visible in blue); however, rabbit anti‐mouse IgG IgG‐Fab/Ab (MP Biochemicals) was employed as the nonspecific background control.^7^, ^9^, ^28^, ^30^, ^49^, ^50^

For the histomorphometric measures and analyses of the signals detected as digitally quantitative expressions on the dual‐IHC immunostained cells, total numbers of positively stained cells on bone surfaces labeled for CD11c^+^/in brown, TRAP^+^/in blue, or both (clearly visible in brown and blue overlaps) were quantified. Briefly, 15–17 randomly selected fields of 15x serial hind limbs’/joints’ sections were employed for the scanning and digital quantification analyses under ×400 magnification via a Carl‐Zeiss Inverted‐Microscope/Axioskop‐40/RS‐Photometrics accompanied with a digital camera on the motorized stages and software as described.^7^, ^9^, ^28^, ^30^, ^42^, ^50^, ^53^ The mean of total numbered TRAP^(+)^ CD11c^(+)^ double‐positive cells (clearly visible brown and blue overlaps) versus single‐positive cells (e.g., visible CD11c^+/^in brown or TRAP^+^/in blue) and the total joints areas of the eroded subchondral/bone versus tissue/perisynovium per mouse on the histological sections were then digitally calculated (~27.5% total surfaces ≈30 mm^2^ area) after subtracting the detected averaged control background signals which were quantifiably described.^7^, ^9^, ^28^, ^30^, ^41^, ^42^, ^49^, ^50^, ^53^ Separately, in parallel, the perisynovial tissues with cellular/inflammatory infiltrates in hind limbs’/joints’ sections (i.e., bone vs. tissue) per mouse samples were also included for quantitation by the histomorphometric analyses, where 15x sections per mouse (for ≥5–7 mice/group) were used for the quantitative analyses. These results are statistically represented as mean ± *SE* per mouse/group, from at least 4–5 sets of repeated experiments, collectively. To quantify the total surface areas of resorptive spots in the digitized scanned fields of all cases from experiments, cells were stripped off using 1 N NaOH for 14–16 h, after which the images of all eroded bone surfaces per field/section were captured as previously described.^7^, ^9^, ^28^, ^30^, ^41^, ^42^, ^49^, ^50^, ^53^

### 2.3 Statistical analyses

For the digital histomorphometric analyses via the quantitative immunostaining of IHC and bone loss/erosion measurements to compare the differences between groups for comparable significance, statistical analysis was performed using the two‐sided Student *t*‐test via the IBM computing software SPSS Statistics (SPSS 22, IBM Corp), and the differences between groups were considered significantly different with >95% confidence, when *p*‐value was <.05.

## 3 RESULTS

### 3.1 Measures of the hind limbs/joints clinical swelling detected in the BM‐chimeras in vivo

We have recently described the basic characterization of T6KO‐null BM chimeras established, which compatibly recapitulated the original TRAF6^(−/−)^‐KO mice reported, lacking the endogenous Mo/Mϕ‐derived classical OCs associated with osteoclastogenesis and bone remodeling, accompanied by slight traces of TRAF6^(−/−)^CD11c^+^ versus CD11b^+^mDCs (i.e., <2% less of donor‐CD45.2/host‐congenic‐CD45.1 ratio) as supported by the FACS analyses post‐BM reconstitution at week 6 for the chimerism yielded.^49^, ^50^ In parallel, there were comparably more clinical swelling and bone loss/erosion in the hind limbs’/joints’ of “CC‐II‐immunized” WT_bmChi and T6KO_ bmChi chimeras detected largely between days 27 and 33 (of weeks 4–5), as compared to those of WT_bmChi‐PBS and T6KO_bmChi‐CC‐II+anti‐TGFβ‐Ab in vivo, suggesting that T6KO_bmChi chimeras can be applied to assess the activities or as effector(s) of specific cell types/lineages (i.e., CD11c^+^mDCs/mDDOCp) in vivo, which was quantifiably IHC measured via labelling of “double‐positive” myeloid‐CD11c^+^TRAP^+^DC‐like multinucleated OCs (i.e., TRAF6^(−/−)^‐mDDOCp) in tissue sections of the joints/paws analyzed. Thus, these above results substantively established the feasibility of TRAF6^(−/−)^‐null chimera generated.^50^ Importantly, such notion was fully supported by TGF‐β neutralization in T6KO_bmChi‐CC‐II+anti‐TGFβ‐Ab^−^ip chimeras tested, where significantly robust reduction in tissue inflammation and the parallel bone loss/erosion were detected, compared to those without neutralization in vivo as reported recently.^50^

Further to the above, in vivo administrations of cytokine or/and anticytokine Ab were applied to the CC‐II‐immunized WT_bmChi and T6KO_bmChi chimeras for re‐assessing the subsequent influence over the levels erosion in/erosion in hind limbs’/joints’ samples harvested in vivo. To this end, there were significant tissue swelling in both WT_bmChi‐CC‐II [as (+)‐control] and T6KO_bmChi‐CC‐II chimeras, from approximately days 27 to 35 (of weeks 4−5; refer to the Figure S1 for the results), in concordance with our prior report^50^; though the clinical onset, progression and the kinetics/timing detected were slightly different from those reported in the DBA‐1 mice (H‐2^q^; a strong responder to CC‐II challenge) and other strains analyzed previously.^9^, ^42^, ^51^, ^52^

Collectively, present findings of the CC‐II‐immunized clinical tissue swelling and progression detected supported the validity of TRAF6^(−/−)^‐null chimeras, thereby prompting us to examine whether myeloid‐CD11c^+^TRAP^+^DC‐like OCs are indeed involved in an alternative pathway of osteoclastogenesis for arthritic bone loss, in which we proposed for its direct contribution in vivo with attributable significance, without the influence of Mo/Mϕ‐derived classical OCs, as depicted in the Figure S1 results and Figure 1 flow chart.

### 3.2 CD11c^+^TRAP^+^DC‐like OCs were clearly and significantly detected in the inflamed hind limbs/joints and eroded bone surfaces of CC‐II‐immunized T6KO_bmChi chimeras in vivo

**Figure 2:**
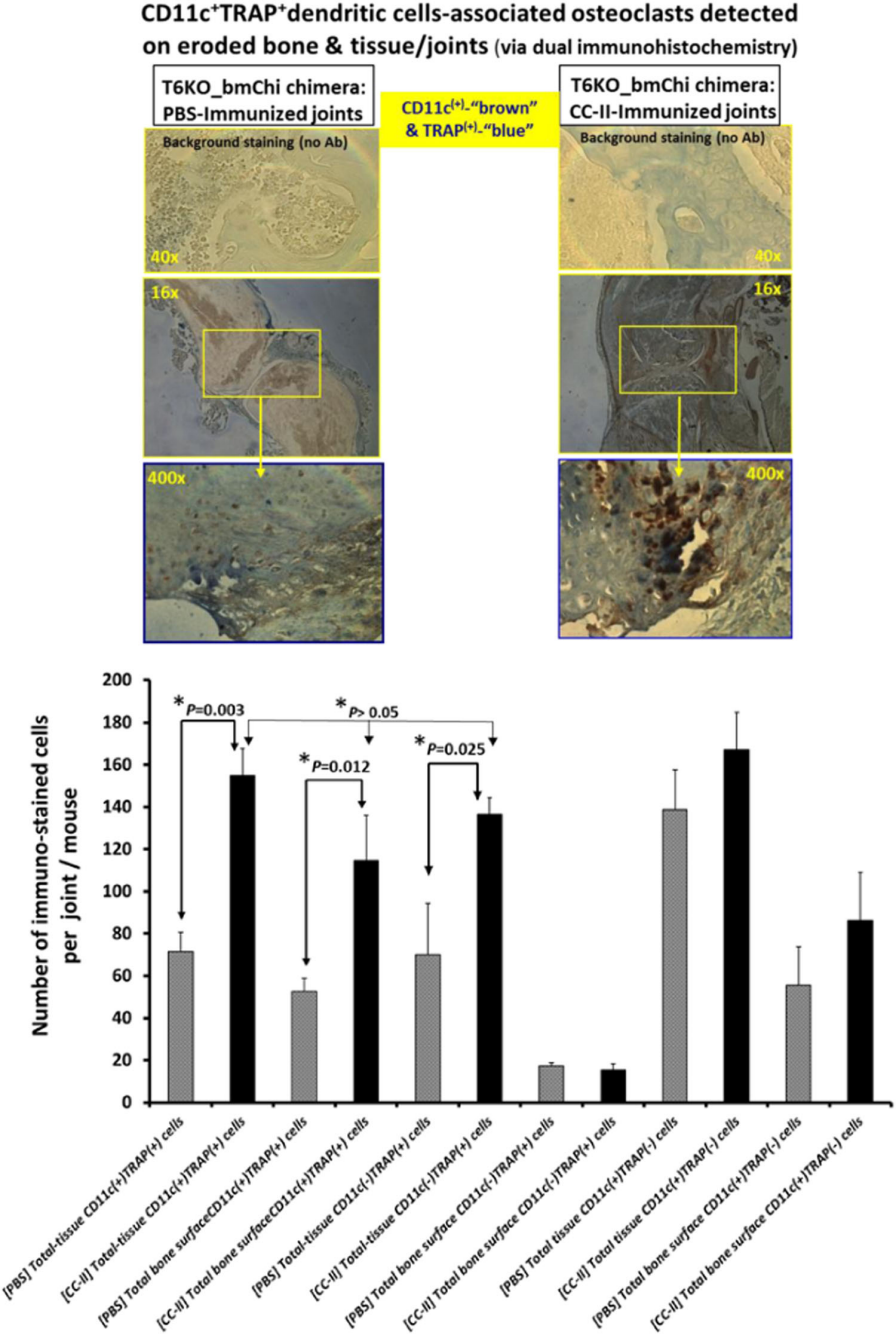
CD11c^+^TRAP^+^DC‐like OCs were significantly and clearly detected in tissues of the inflamed hind limbs/joints and eroded bone surfaces of CC‐II‐immunized T6KO_bmChi chimeras in vivo. Legend: (upper panel). Based on IHC staining of sections prepared from ≥15 serial hind limbs’/joints’ tissues per mouse samples (i.e., typical areas of the distal tibia or tibiotalar/calcaneum harvested from day 0 to week 6), the resultant representatives depicted that there were much more double‐positive myeloid CD11c^+^ TRAP^+^‐multinucleated (≥2–3 nuclei) OC‐like cells with distinguished CD11c^+^cells/in brown and TRAP^+^cells/in blue detected, in control PBS (top/left‐sided middle and lower images) and CC‐II‐immunized chimeras (top/right‐sided middle and lower images) under ×16 to ×400 magnifications; the background staining without Ab used were at the top/left versus top/right images as depicted (lower panel). There were more quantified double‐positive CD11c^+^TRAP^+^OC‐like cells detected in both total tissue/synovial versus bone‐surface areas (i.e., sub‐chondral erosion) in CC‐II‐immunized than those in PBS‐immunized mice (*p* = .003 vs. *p* = .012, respectively); labeled from the left to right as [PBS] total tissue/bone surface CD11c^+^TRAP^+^cells [CC‐II], total‐tissue/bone surface CD11c^+^TRAP^+^cells, and others [PBS or CC‐II] being CD11c^(−)^TRAP^(+)^ versus CD11c^(+)^TRAP^(−)^ single‐positive cells, etc., where the inflammatory infiltrates and bone erosion were more significantly detected in the hind limbs/joints of C57BL/6 mice. The above results were derived from four independent experiments with 5–7 mice/group/set. The Student *t*‐test and the differences between groups were considered significantly different with >95% confidence when the *p*‐value was <.05. DC, dendritic cell; OC, osteoclast; PBS, phosphate‐buffered saline.

Interestingly, it was found via quantitative dual IHC with digital histomorphometry that (i) there were enumerable double‐positive myeloid CD11c^+^TRAP^+^OC‐like multinucleated (≥2–3 nuclei) cells detected (Figure 2, upper panel: visibly distinguishable CD11c^+^ cells in brown and TRAP^+^ cells in blue), in representative tissues/bone samples harvested from hind limbs/joints of the PBS and CC‐II immunized WT mice, and (ii) there were significantly more quantifiable CD11c^+^TRAP^+^OC‐like cells detected and directly observed in the histological sections analyzed from both total tissue/synovial versus eroded/bone surface areas in hind limbs of the CC‐II‐immunized mice than those in the control PBS‐immunized ones (Figure 2, lower panel/bar diagrams to the left; *p* = .003 vs. *p* = .012, respectively; labelled as: [PBS] total tissue/bone surface CD11c^+^TRAP^+^ cells and [CC‐II] total tissue/bone surface CD11c^+^TRAP^+^ cells), where the tissue inflammation and bone loss mimicking the obvious inflamed joints did take place comparably in the WT‐C57BL/6 strain (see Figure 4 below), despite being a poor responder to CC‐II challenges like other reports described elsewhere.^50^, ^51^, ^52^ Further, there were more quantifiably single‐positive TRAP^(+)^CD11c^(−)^ cells physically detected in the total tissue/synovium of CC‐II‐immunized mice than those in PBS‐immunized ones (*p* = .025; see Figure 2); however, this difference was not apparent in the bone surfaces of both groups. Meanwhile, variable numbers of CD11c^(+)^TRAP^(−)^ cells detected were present throughout the sections of total tissue versus bone surfaces areas. Notably, such TRAP^(+)^CD11c^(−)^ versus CD11c^(+)^TRAP^(−)^ single‐positive cells present in total tissues likely belonged to the tissue versus synovial Mo/Mϕ subsets residing in situ that were activated or upon being activated under different steady‐state or/and inflammatory conditions in the local environment (i.e., to CC‐II & PBS^50^, ^51^). The increased numbers of CD11c^(+)^TRAP^(−)^‐single‐positive cells co‐localized near or at the eroded bone surfaces of CC‐II‐immunized mice, in contrast to those in control PBS‐immunized ones, may be associated with slight traces of the juxta‐tissue bound/co‐localized granulocyte‐macrophage progenitors (GMPs), erythro‐myeloid progenitors (EMPs), or macrophage‐OC‐DC progenitors (MODPs; being CD11b^−^CD31^+/hi^Ly6C^−/+^C‐kit^+^), Mo‐derived CD11c^(+)^DCs, bona fide CD11c^(+)^TRAP^(−)^DCs in situ, or/and plasmacytoid DC of the GMP lineage^18^, ^31^, ^32^, ^33^, ^43^, ^44^ that were silenced or not properly tuned (~TRAP^(−)^) to manifest OC‐like phenotype during inflammatory conditions in vivo (see Section 4 and Figure 5—the proposed pathways illustrated).

### 3.3 Double‐positive CD11c^+^TRAP^+^OC‐like cells were detected in T6KO‐bmChi‐CC‐II chimeras lacking

#### 3.3.1 Mo/Mϕ‐derived classical OCs manifest significant bone loss in hind limbs/joints at week 6 in vivo

**Figure 3:**
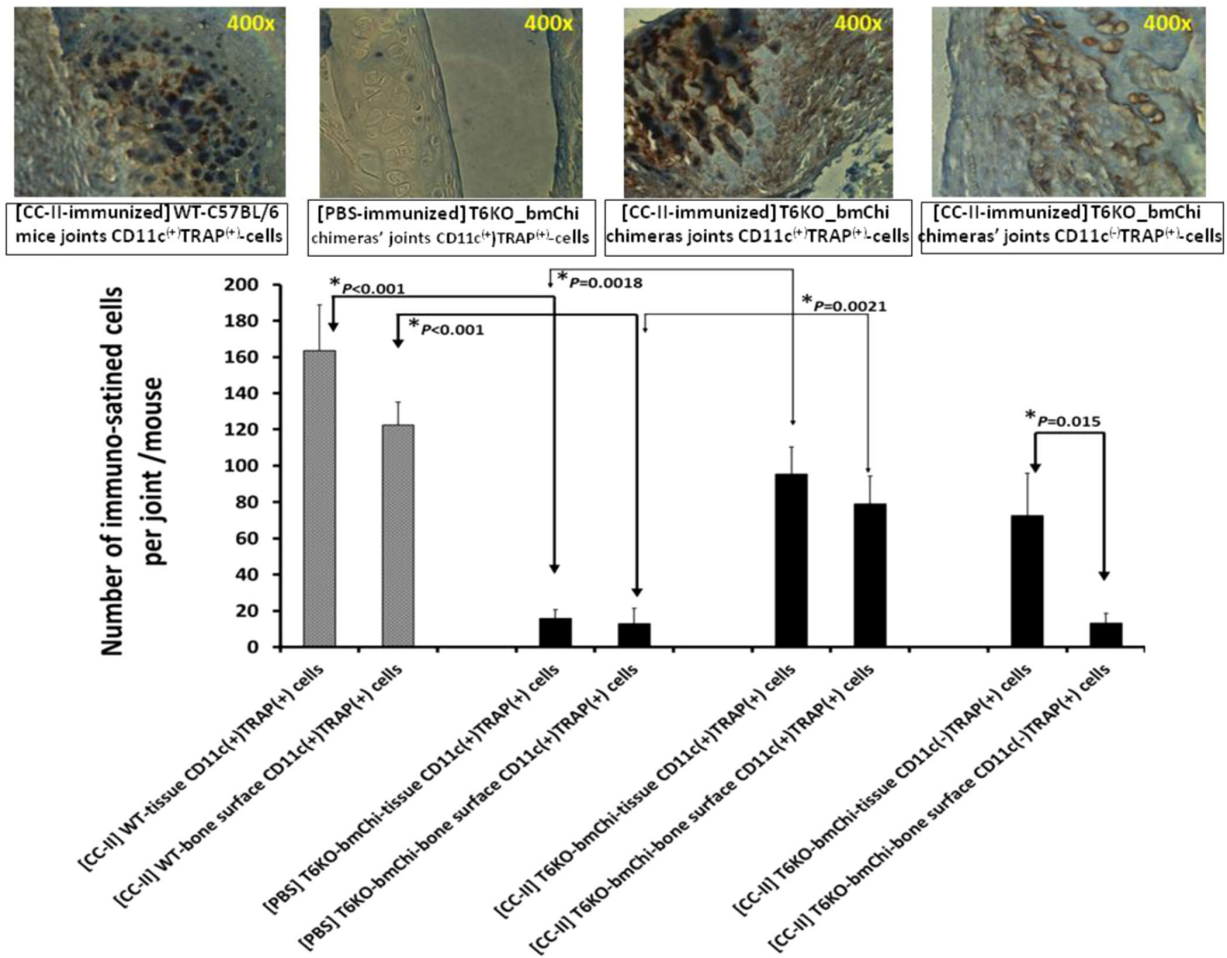
CD11c^+^TRAP^+^OC‐like double‐positive cells which were detected in T6KO‐bmChi‐CC‐II chimeras lacking Mo/Mϕ‐derived classical OCs manifest significant bone loss in the hind limbs/joints at week 6 in vivo. Legend: (upper panel: from left to right) There were significantly more quantifiable double‐positive CD11c^+^TRAP^+^DC‐like OCs (≥2–3 nuclei) detected in the tissue and bone surfaces of CC‐II‐immunized WT mice, the (+)‐control labeled as [CC‐II immunized] WT mouse joint CD11c^(+)^ TRAP^(+)^ cells, in contrast to those much fewer CD11c^+^TRAP^+^DC‐like OCs detected in tissue and bone surfaces of PBS‐immunized T6KO_bmChi chimeras, labeled as [PBS immunized] T6KO‐bmChi mouse joint CD11c^(+)^ TRAP^(+)^cells (both *p* < .001; respectively). Conversely, there were much more CD11c^+^TRAP^+^ double‐positive/DC‐like OCs detected in both synovial tissues and bone surfaces of the joints in CC‐II‐immunized T6KO_bmChi chimeras labeled as [CC‐II immunized] T6KO‐bmChi mouse joint CD11c^(+)^TRAP^(+)^cells, when compared to those detected in their counterparts of PBS‐immunized T6KO_bmChi chimeras (*p* = .0018 vs. *p* = .021, respectively). Also, significantly more TRAP^(+)^CD11c^(−)^ single‐positive mononuclear cells were detected in tissues than bone surfaces (*p* = .015) of CC‐II‐immunized T6KO_bmChi chimeras, labeled as [CC‐II immunized] T6KO‐bmChi mouse joints/TRAP^(+)^CD11c^(−)^ cells, where the representative sections showing notable tissue/synovial mononuclear cells in situ. DC, dendritic cell; OC, osteoclast; WT, wild type.

To this end, we employed the established method of lethal irradiation and BM reconstitution to generate mature adult T6KO‐bmChi chimeras lacking the Mo/Mϕ‐derived classical OCs, with which the contributions or/and role of myeloid‐CD11c^+^DCs, as OCps, in relation to yielding CD11c^+^ TRAP^+^DC‐like OCs with joints’ tissue inflammation and associated bone loss post‐CC‐II immunization (see Figure 3, upper panel), in comparison to those detected in CC‐II‐immunized WT mice [as (+)‐control], can be evaluated in vivo. The results (Figure 3, upper/lower panels) showed that (i) there were only background levels of double‐positive CD11c^+^TRAP^+^DC‐like OCs observed and detected in the tissues (*p* < .001) and bone surfaces (*p* < .001) of PBS‐immunized T6KO_bmChi‐DC mice, in contrast to those detected abundantly and significantly in CC‐II‐immunized WT mice [as (+)‐control] via statistical comparisons; (ii) conversely, there were much more enumerated double‐positive CD11c^+^TRAP^+^DC‐like OCs physically detected in the hind limbs’/joints’ tissues (*p* = .0018) and bone surfaces (*p* = .0021) of CC‐II‐immunized T6KO_bmChi‐DC mice, as opposed to those detected in CC‐II‐immunized mice’ counterparts instead. These data strongly suggested that myeloid CD11c^+^DCs were closely associated with the presence of double‐positive CD11c^+^TRAP^+^TRAF6^(−/−)^DC‐like OCs physically located in both inflamed tissues and eroded bone surfaces of CC‐II‐immunized T6KO_bmChi‐DC mice, lacking the Mo/Mϕ‐derived classical OCs in vivo (Figure 3 legend). Meanwhile, some significant numbers of TRAP^(+)^CD11c^(−)^‐“single‐positive” cells were detected mainly in hind limbs’/joints’ tissue sites (*p* = .015) rather than those in bone surfaces of the T6KO_bmChi‐CC‐II mice, suggesting that some synovial associated tissue‐bound Mo/Mϕ subset(s) may have been activated under inflammatory environments in situ, consistent with the well‐described phenomenon reported in the past.^13^, ^18^, ^19^, ^38^, ^39^, ^40^, ^43^, ^44^, ^45^

### 3.4 The resultant IL‐17 administration was associated with compatibly detected CD11c^+^TRAP^+^ TRAF6^(−/−)^DC‐like OCs, which correlated well to the inflammatory bone loss/erosion upon TGF‐β neutralization in the immunized T6KO_bmChi‐CC‐II chimeras by week 6 in vivo

**Figure 4:**
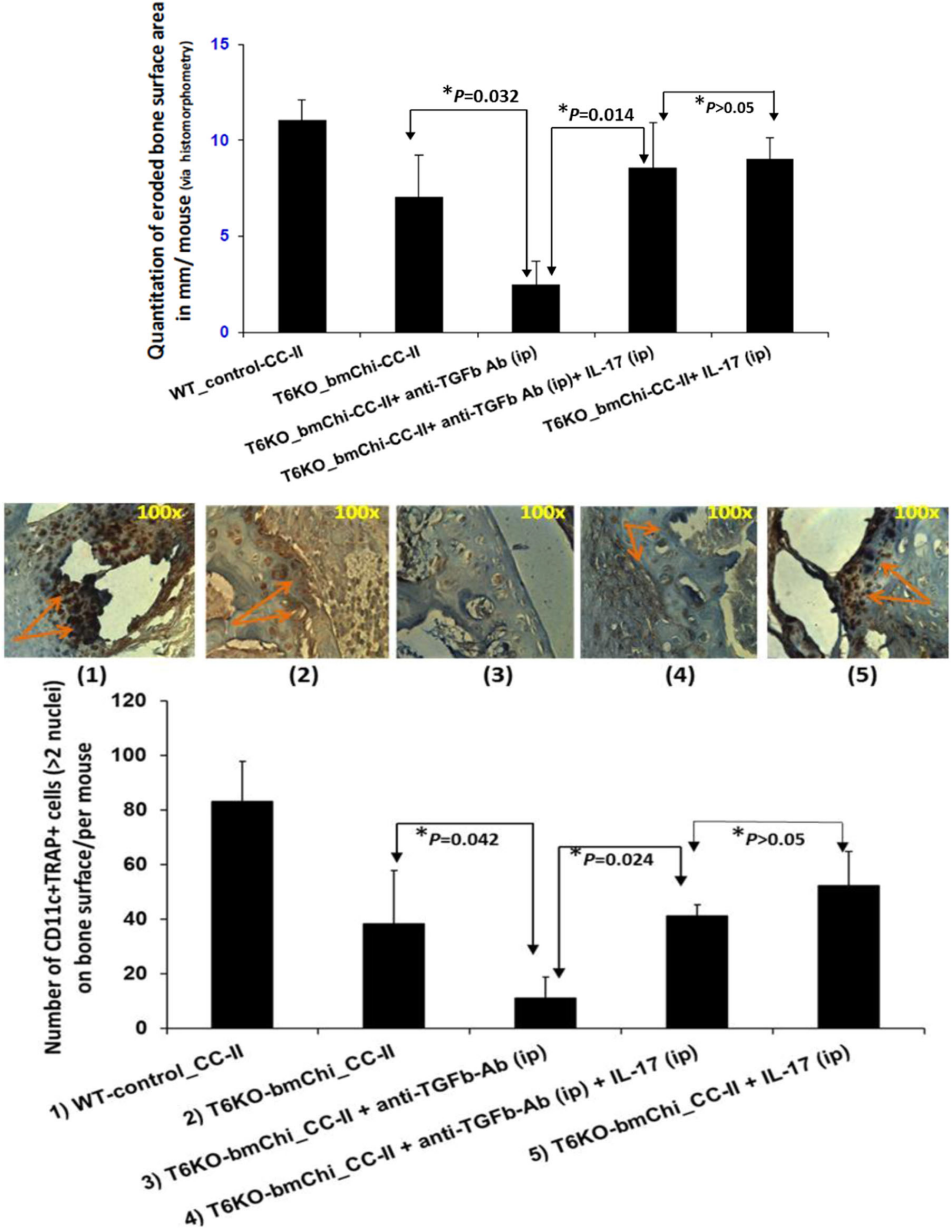
The IL‐17 administration was associated with compatibly detected CD11c^+^TRAP^+^TRAF6^(−/−)^. DC‐like OCs, which correlated well to the inflammatory bone loss in the hind limbs/joints upon TGF‐β neutralization in the immunized T6KO_bmChi‐CC‐II chimeras by week 6 in vivo. Legend: (upper panel) The quantitative histomorphometrical measures of CD11c^(+)^TRAP^(+)^ double‐positive OCs detected per mouse hind limbs/joints were plotted against each group for statistical analyses as depicted. The CC‐II‐immunized WT mice was set as (+)‐control, labeled as [CC‐II immunized] WT mouse joint (in tissue vs. bone surfaces) CD11c^(+)^TRAP^(+)^ cells. Then, in sequence, CC‐II‐immunized T6KO_bmChi chimeras, labeled as [CC‐II immunized] T6KO‐bmChi mouse joint CD11c^(+)^TRAP^(+)^cells (in tissue vs. bone surfaces), compared to those detected in their counterparts of PBS‐immunized T6KO_bmChi chimeras (*p* = .0018 vs. *p* = .021, respectively). For comparison to other groups in the right end, single‐positive TRAP^(+)^CD11c^(−)^ cells were detected more in synovial tissues than in bone surfaces (*p* = .015) of CC‐II‐immunized T6KO_bmChi chimeras labeled as [CC‐II immunized] T6KO‐bmChi mouse joint/TRAP^(+)^CD11c^(−)^ cells, showing notable tissue/synovial mononuclear cells in situ. The above results shown were from five independent experiments with five mice per group/set. *Note*: Other controls (i.e., PBS injected into WT mice transferred with WT‐BM [as WT_bmChi‐PBS] and PBS injected into WT mice transferred with TRAF6 KO‐BM [as T6KO_bmChi‐PBS] reported recently^50^ and, thus, omitted herein). (Middle panel) There was significantly less bone loss detected in T6KO_bmChi‐CC‐II chimeras having received TGF‐β neutralization by week 6 when compared to those without [*p* = .032]. In addition, exogenous rm‐IL‐17A administration without TGF‐β neutralization manifested no significantly increased bone loss detected in the CC‐II‐immunized T6KO_bmChi‐CC‐II + IL‐17‐(ip) chimeras (*p* > .05). *Note*: The isotypic‐control Ab applied did not affect the resultant CD11c^**+**^ TRAP^(+)^‐DC‐like OCs detected and the bone surface areas/mm^2^ quantified and thus were omitted herein. (Lower panel) For the quantitative histomorphometry of CD11c^+^TRAP^+^DC‐like double‐positive OCs detected in tissue samples of the hind limbs/joints, the results showed equivalently similar inflammatory bone loss measured in tissues and bone surfaces in IL‐17‐treated and CC‐II‐immunized T6KO_bmChi chimeras, with or without TGF‐β neutralization (*p* = .042 & *p* = .024, respectively), in relation to those depicted in Figure 4A—upper panel; whereas, rm‐IL‐17A in vivo/ip‐administration alone yielded no significant effects on subsequent bone loss detected in immunized T6KO_bmChi‐CC‐II chimeras (*p* > .05) in vivo. *Note*: Other controls (i.e., PBS injected into WT mice transferred with WT‐BM [as WT_bmChi‐PBS] and PBS injected into WT mice transferred with TRAF6 KO‐BM [as T6KO_bmChi‐PBS] reported recently^50^ and, thus, omitted above). DC, dendritic cell; OC, osteoclast; PBS, phosphate‐buffered saline; WT, wild type.

Further, to have examined the joints’ bone loss in hind imbs via PBS & CC‐II Immunization, WT mice immunized with CC‐II were then set as (+)‐control for measuring and comparing subsequent bone loss/erosions in the presence of IL‐17 versus with/without TGF‐β neutralization in vivo^50^ by employing the digital histomorphometrical measures (see Figure 4, upper panel). The results showed that (i) there were significantly less bone loss in the immunized T6KO_bmChi‐CC‐II, upon TGF‐β neutralization, by week 6/day 42, when compared to those without neutralization (*p* = .032; Figure 4, upper panel); (ii) the addition of exogenous IL‐17 significantly up‐restored those with much reduced bone loss detected in T6KO_bmChi‐CC‐II mice having received TGF‐β‐neutralization (*p* = .014), suggesting that cytokine IL‐17 stimulation engaged distinct signaling involved in the CD11c^+^ TRAP^+^TRAF6^(−/−)^DC‐like OC‐mediated inflammatory bone loss, in the absence of TGF‐β in vivo. However, administration of IL‐17 alone was unable to stimulate or engage a comparable influence and/or effector activity “without” TGF‐β neutralization in the local joints’ environment (*p* > .05), indicating that IL‐17 and TGF‐β cross interactions may provide critical interplay signal(s) to immature CD11c^+^mDCs/mDDOCp before developing to “double‐positive”‐CD11c^+^TRAP^+^TRAF6^(−/−)^DC‐like OCs significantly associated with the inflammation‐induced bone loss and osteoclastogenesis in vivo, in the absence of Mo/Mϕ‐derived classical OCs.

**Figure 5:**
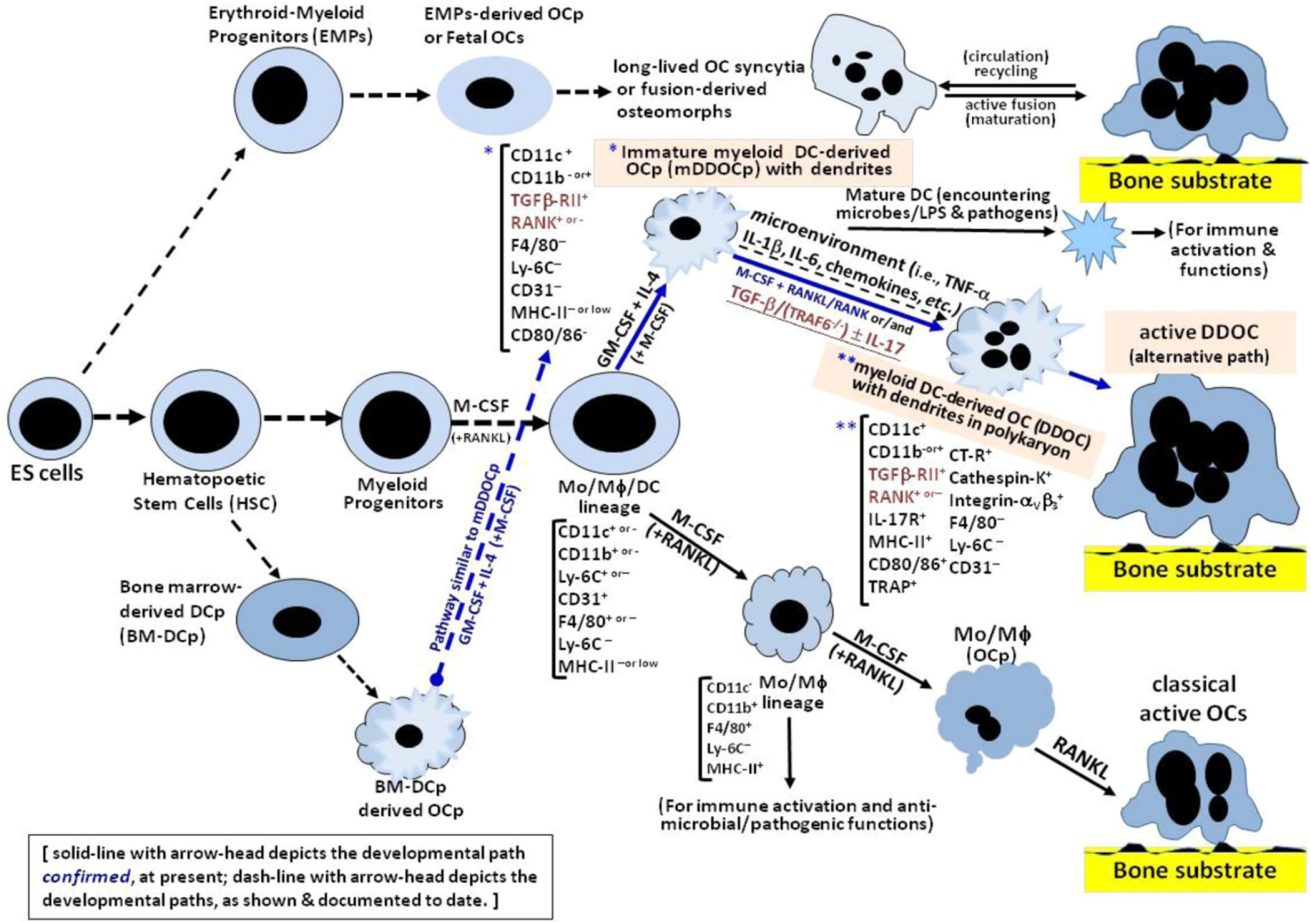
The alternative pathways of mDCs/mDDOCp(OCp)‐associated osteoclastogenesis revisited. DC, dendritic cell.

When the histological sections were assessed by quantitative dual IHC and enumeration of CD11c^+^TRAP^+^DC‐like OCs physically detected in tissue samples of the hind limbs analyzed, the results showed a rather comparable and similar pattern detected (see Figure 4, middle/lower panels) to those observed in the upper panel of Figure 4. In essence, distributions of the enumerated CD11c^+^TRAP^+^DC‐like (≥2–3 nuclei) OCs in the joints samples via quantitation (of tissues/bone surfaces) were both in directly concordance with correspondent “bone loss” levels detected in the IL‐17‐treated T6KO_bmChi‐CC‐II mice with and without TGF‐β neutralization in vivo, respectively (*p* = .042 & *p* = .024; Figure 4, lower panel); however, IL‐17 stimulation alone exerted no such effects to compatibly driving or developing inflammatory bone loss, instead (*p* > .05), consistent with those detected CD11c^+^TRAP^+^TRAF6^(−/−)^DC‐like OCs in parallel. Therefore, to this end, an alternative pathway of mDCs/mDDOCp‐associated osteoclastogenesis is theoretically proposed and theme highlighted for an overall revisitation (see Figure 5 diagram depicted and the discussion below).

## 4 DISCUSSION

Through the dual IHC and quantification of specific DCs subsets studied, the present findings clearly provided the direct physical evidence that CD11c^+^TRAP^+^mDCs‐OCp/DC‐like OCs were significantly involved in driving an alternative pathway for inflammation‐induced osteoclastogenesis in vivo, without the Mo/Mϕ‐derived classical OCs in CC‐II‐immunized TRAF6^(−/−)^‐null chimeras. Meanwhile, IL‐17/IL‐17Rs signaling engages distinctive interplays with CD11c^+^TRAP^+^TRAF6^(−/−)^DC‐like OCs via TGF‐β‐mediated “stepwise” development for subsequent osteoclastogensis and arthritic bone loss, without TRAF6‐mediated signaling or/and intermediates in vivo, which is an uniquely new finding that has not been revealed previously.^5^, ^9^, ^13^, ^14^, ^33^, ^38^, ^39^, ^40^, ^41^, ^42^, ^43^, ^44^, ^45^, ^46^, ^47^ Interestingly, the dual IHC stained CD11c^+^TRAP^+^DC‐like OCs represented the majority of infiltrating immune cells physically detected in the inflamed synovium/bone surfaces of hind limbs of the CC‐II‐immunized WT‐mice, quantitatively indifferent to those TRAP^(+)^CD11c^(−)^‐single‐positive cells (i.e., representing CD11c^(−)^Mo/Mϕ‐derived TRAP^+^OCs) in the tissue counterparts (Figure 2; *p* > .05), which was sought traditionally as the dominant cell types responsibly attributed to inflammatory bone loss/resorption. This finding was consistent with those shown in Figure 3, where CD11c^+^TRAP^+^double‐positive DC‐like OCs detected in the inflamed synovium/bone surfaces represented the majority and most of the local/residential cells comparable to those detected TRAP^(+)^ CD11c^(−)^cells in the T6K_bmChi‐CC‐II chimeras. Moreover, these findings are in high concordance with our previous in vitro and in vivo characterization described, where the mCD11c^+^TRAP^+^DC‐like multinucleated OCs existed^9^, ^42^, ^49^, ^50^ and were compatibly associated with CC‐II‐induced arthritic bone loss detected in DBA‐1 mice, a strong responder strain well documented.^51^, ^52^

The CD11c^+^ expression has highly suggested the representation of mDC lineage in mice.^36^, ^54^ Due to the heterogeneity of myeloid cells, it might not necessarily be delineated exclusively to DCs or/and Mo/MQ lineages thought:^8^, ^19^, ^31^ despite other studies favor strongly their direct correlations in vivo.^36^, ^54^, ^55^, ^56^ For instance, CD11b^+^Mo‐lineaged cells may represent a very minor fraction of OCp subset,^8^ whereas BM‐derived CD11b^+^DCs are not osteoclastogenic in nature: in contrast to DCs bearing CD11b^(low or −/−)^ manifest rather high osteoclastogenic activity.^8^, ^32^, ^33^, ^38^, ^40^, ^43^ Studies had shown that a separate subset of CD11c^+^Mo‐lineaged cells with DC‐like phenotypes may develop into the classical/conventional DCs upon transmigration through the endothelium^56^, ^57^; meanwhile, CD11b expression becomes downregulated as OCps develop into active OCs.^43^, ^54^ To this context and extent, we cannot absolutely exclude that there is no traceable minor contaminant(s) of “Mo‐lineage” DC‐like multinucleated OCps co‐localized in/around the synovial tissues of such inflammatory foci (i.e., tissue/synovial Mϕ being TRAP^(+)^CD11c^(−)^‐as “single‐positive” cells) which existed in Figures 2 and 3 [also: 1–2,38–40] associated with bone loss detected in the TRAF6‐null chimeras. Conceivably, it's trustworthy to mention that minor contaminant(s) did not likely account for the robust bone loss detected and yielded from double‐positive CD11c^+^TRAP^+^ TRAF6^(−/−)^DC‐like OCs analyzed in the CC‐II‐immunized hosts in vivo (see Figures 3 and 4); we had previously reported that (i) only committed mDCs carry CD11c^+^ expression in BM‐derived DCs,^7^, ^9^ (ii) Mo/MQ‐depletion does not deviate mDCs‐associated OCp development in vitro,^7^, ^9^, ^42^ and (iii) almost all committed mDC postactivation manifest TRAP^+^‐CD11b^**−**^CD11c^+^‐multinucleated OC phenotype, thus confirming such CD11c^+^OCp status.^7^, ^9^, ^42^ Moreover, the resulting TGF‐β and IL‐17 interactions (Figure 4) were comparable with our in vitro study reported recently,^49^ suggesting a distinctive stepwise development from mDCs/mDDOCp to the osteoclastogenic pathway.^49^, ^50^

The present findings suggest that, even if minor contaminant(s) from other OCp subset(s) did exist, it is highly un‐probable to play a significant role to developing the CD11c^+^TRAP^+^DC‐like OCs responsible for inflammatory bone loss in the current mouse model studied (Figures 2, 3, 4). Prospectively, one limitation of the present study is yet to be performing the tracing analyses via molecular beacon‐tagged CD11c^+^mDCs cells in vivo throughout their time course activity with parallel kinetics as designed in Figure 1, which will delineate and confirm the origin and ultimate fate(s) of CD11c^+^TRAP^+^DC‐like OCs that are one progeny(s) from the mDCs/OCp lineage development. Overall, based on recent studies where the diverse developmental lineages (i.e., EMPs, GMPs, etc.^18^, ^31^, ^32^, ^33^, ^43^, ^44^, ^58^, ^59^) may diverge individually before converged to final osteoclastoegnic pathways associated with the homeostatic versus pathogenic bone remodeling, in addition to the traditional theme of the Mo/Mϕ‐lineage classical OCp cells, which is now theoretically illustrated in Figure 5 as depicted for a summation.

Despite the TRAF6‐independent osteoclastogenesis had been prior reported,^20^, ^21^ however, its developmental ontogenesis and interactions remained unclear. Moreover, the resulting TGF‐β and IL‐17 interactions (Figure 4) were comparable with those in our in vitro study,^49^ suggesting a distinctive stepwise development from mDCs/mDDOCp to the osteoclastogenic pathway.^49^, ^50^ Such stepwise twist‐in‐turns alternative pathways may typically involve either influx of the precursors’ egress from circulations or/and recruitments of scout‐typed mDCs/OCp subsets in situ under proinflammatory conditions in response to local calls via juxtacrine or chemotactic signaling.^10^, ^13^, ^16^, ^33^, ^36^, ^48^, ^57^, ^60^ Paradoxically, JAK/STAT‐mediated SOCS3‐signaling pathway was reported having been affected during CD11c^+^mDC transition to TRAP^(+)^ OCp for osteoclastogenesis, regardless of the TRAF6 expression levels.^53^ It remains to be determined why such differential regulations may exist; intriguingly, how does IL‐17 versus TGF‐β signal interactions with mDDOCp/mDCs subset develop under the homeostatic or pathologic prospect for osteoclastogenesis, and bone remodeling in vivo will require further study to reveal the underlying causes.^13^, ^14^, ^17^, ^49^, ^50^, ^61^, ^62^, ^63^

## 5 CONCLUSION

The present study confirms our proposed past notion that the immature mCD11c^+^DCs/mDDOCp that manifest precursor phenotype(s), acting as OCp, can develop into active and functional OCs for inflammation‐induced osteoclastogenesis, even in the absence of Mo/Mϕ‐derived classical OCs in vivo, where such a stepwise TGF‐β‐mediated regulation of mDCs/OCp studied above provides an unrecognized novel insight of the underlying osteo‐immune interactions; i.e., IL‐17 and TGF‐β mediated distinct cross talk with TRAF6^(−/−)^CD11c^+^mDCs/mDDOCp present in the environmental milieu sufficient to compatibly driving bona fide alternative pathway of osteoclastogenesis (Figure 5). Such (non)‐discriminative twist‐in‐turns osteo‐immune interactions will require more study into the molecular insights to decipher its physiologic sequelae versus impact via in vivo models and analogies addressed to the human conditions, including arthritic, periodontal, or/and osteoporotic disorders.

## AUTHOR CONTRIBUTIONS

Yen Chun G. Liu and Andy Yen‐Tung Teng were involved in writing—original draft & revisions. Yen Chun G. Liu and Andy Yen‐Tung Teng were involved in writing—review & editing. Yen Chun G. Liu was involved in the study designs, data acquisition, first draft of the manuscript write‐up, analyses, and revisions of the figures and the manuscript. Andy Yen‐Tung Teng was involved in all aspects of the study design, protocols, establishments, and its modifications along with discussions for analyses, interpretations, and the overall issues of the entire project. All authors have read and agreed to the published version of the manuscript.

## CONFLICT OF INTEREST STATEMENT

The authors declare no conflicts of interest.

## ETHICS STATEMENT

The present project, involving the lab animals, was conducted according to the guidelines for animal protection, welfare, and use, which were approved for protocol by the Institutional Animal Care & Use Committee, with IACUC accession numbers (#98017 & #98183), Kaohsiung Medical University, Kaohsiung City, Taiwan.

## Supporting information

Supporting information.

Supporting information.

## Data Availability

The authors declare that the data supporting the present findings of this article are available from the authors upon reasonable request.
