# Supplementary material for: Distinct cross talk of IL‐17 & TGF‐β with the immature CD11c+TRAF6(−/−)‐null myeloid dendritic cell‐derived osteoclast precursor (mDDOCp) may engage signaling toward an alternative pathway of osteoclastogenesis for arthritic bone loss in vivo
Source: Immun Inflamm Dis. 2024 Feb 8;12(2):e1173. doi: 10.1002/iid3.1173 (PMC10851637; doi:10.1002/iid3.1173)
Supplement: Supplementary file 1 — Supporting information. [file IID3-12-e1173-s001.pdf]

Five figures & one Supplementary-data-of-Fig-1:

[Article title: Distinct cross-talk of IL-17 & TGF-β with the immature CD11c⁺TRAF6$^{(-/-)}$-null myeloid dendritic cell-derived osteoclast precursor (mDDOCp) may engage signaling towards an alternative pathway of osteoclastogenesis for arthritic bone loss *in-vivo*. *by Yen Chun G. Liu & Andy Yen-Tung Teng*]

Figure 1: Flow-chart of the experimental protocols

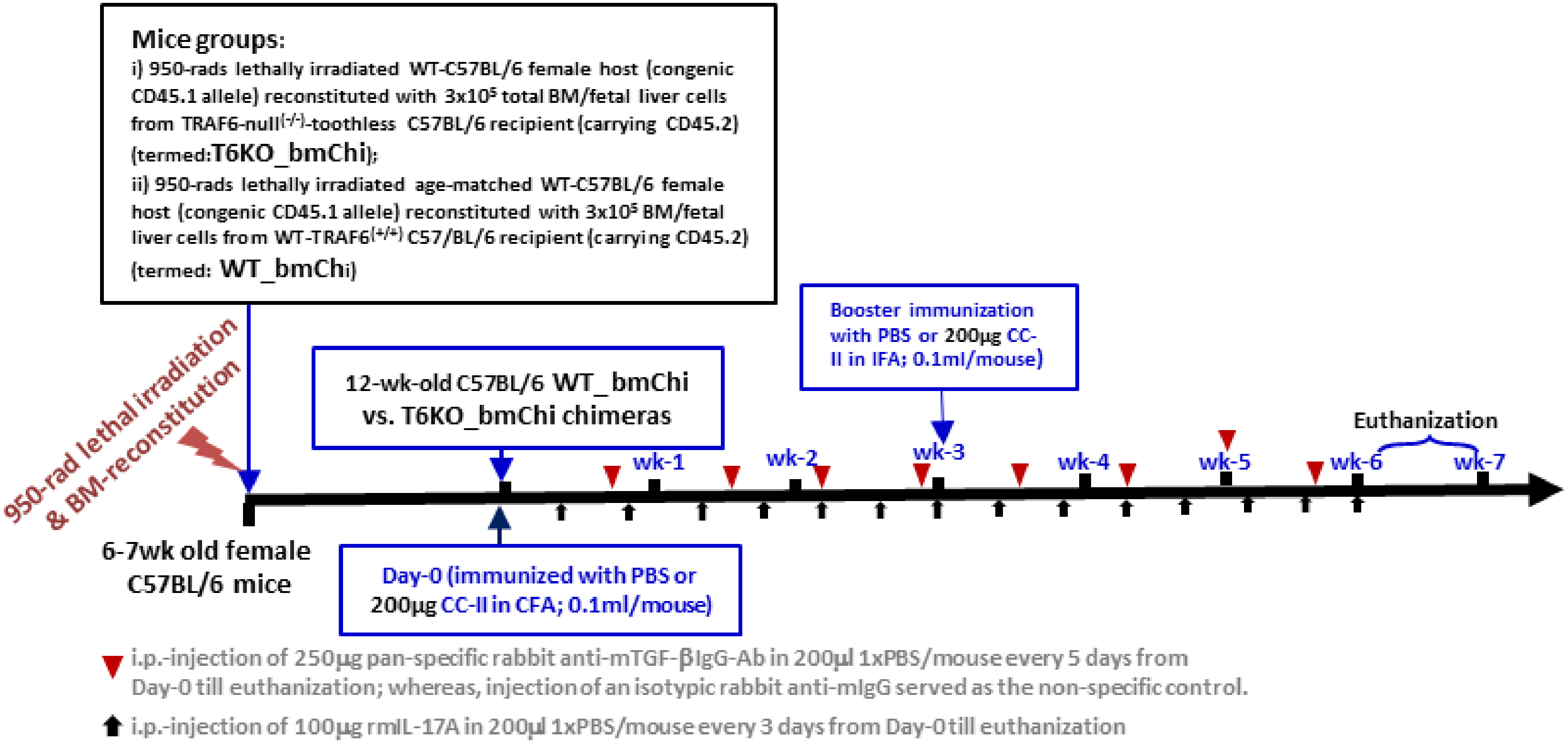

Fig 2 **(upper & lower panels):**

CD11c+TRAP+DC-like OCs were significantly and clearly detected in the inflamed joints/ tissues and the eroded bone -surfaces of CC-II-immunized T6KO_bmChi chimeras *in vivo.*

CD11c+TRAP+dendritic cells-associated osteoclasts detected on eroded bone & tissue/joints (via dual immunohistochemistry)

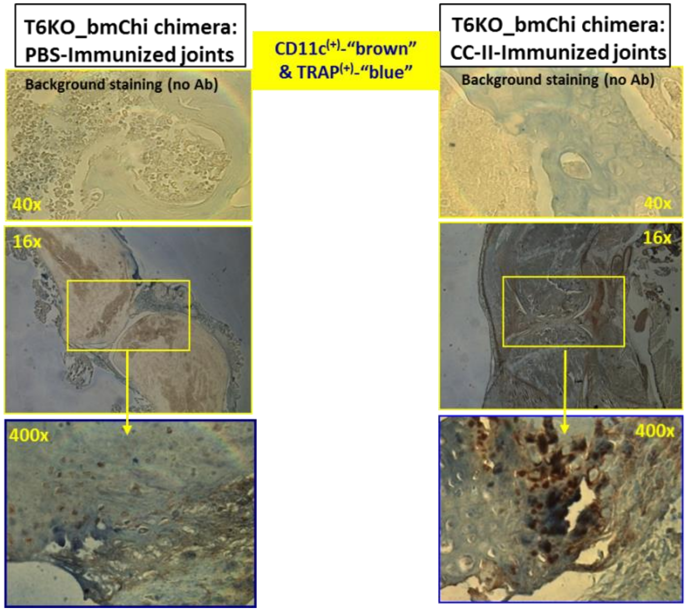

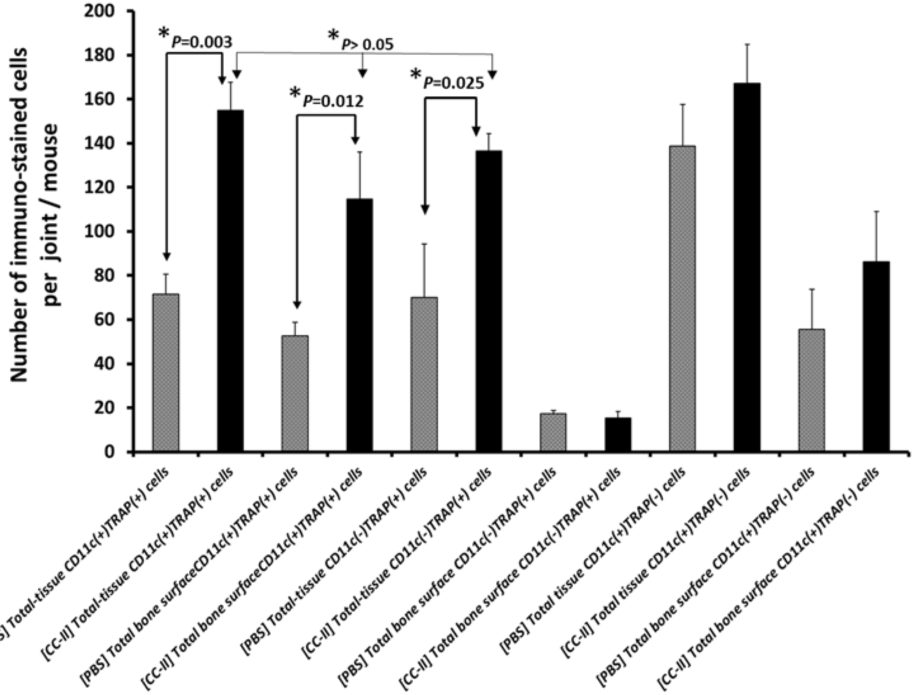

Fig. 3 (upper & lower panels)

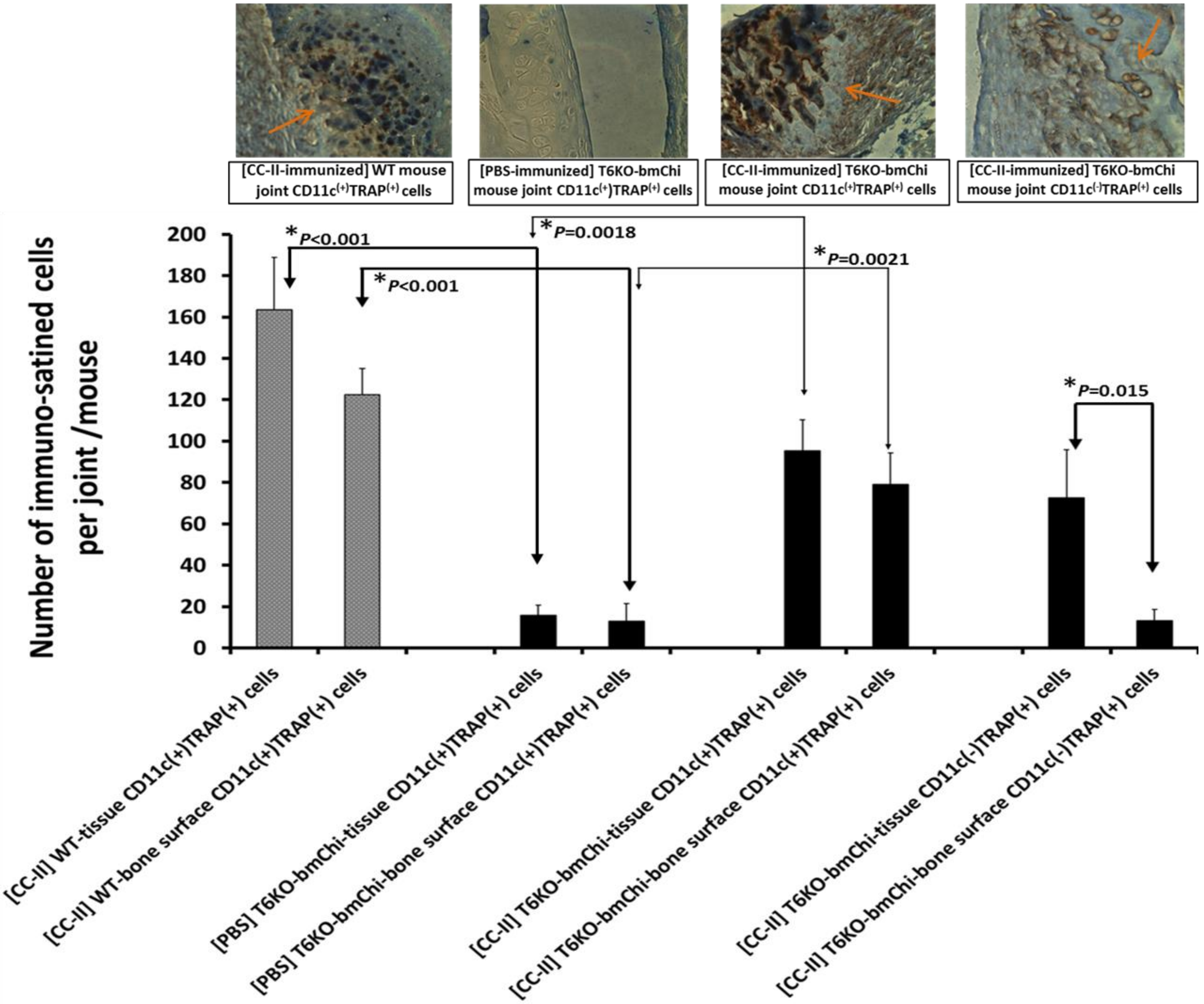

Fig. 4 (upper panel)

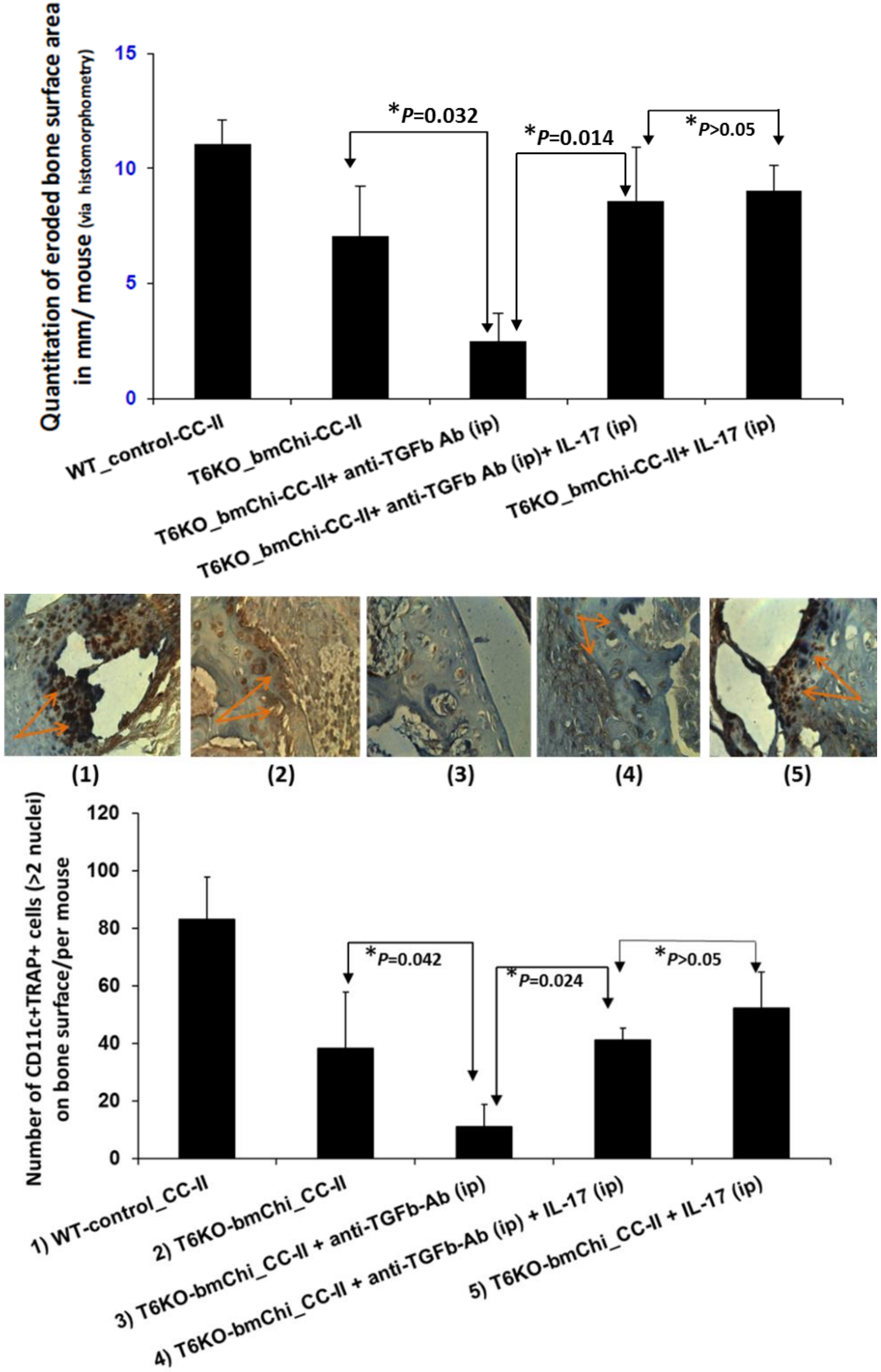

Fig. 5: The proposed alternative pathways of mDCs/mDDOCp(OCp)-associated osteoclastogenesis revisited

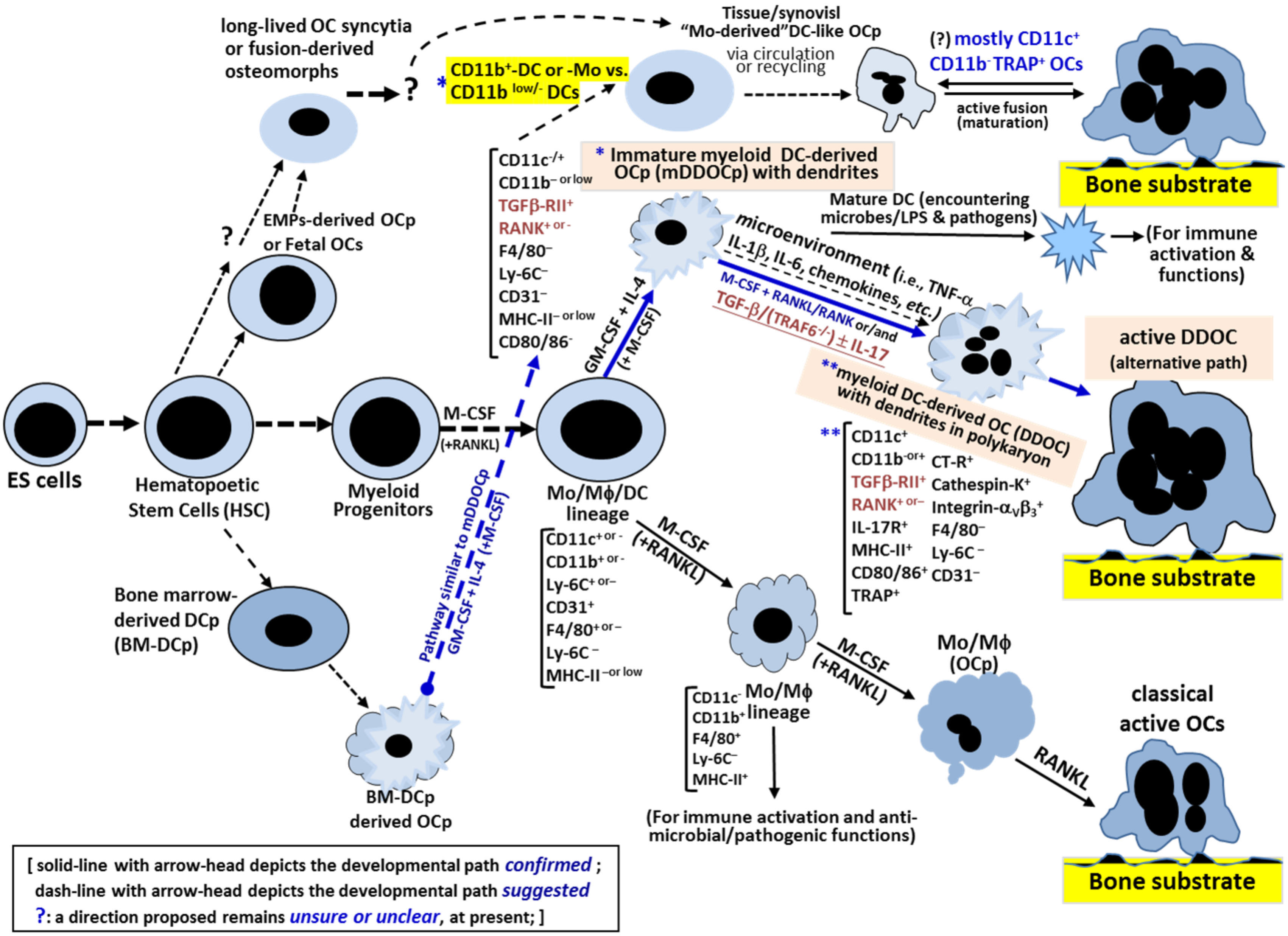

Supplementary-Fig. 1: Significant swelling of the joints/paws detected in CC-II-immunized WT_bmChi & T6KO_bmChi chimeras at wk-6 *in vivo.*

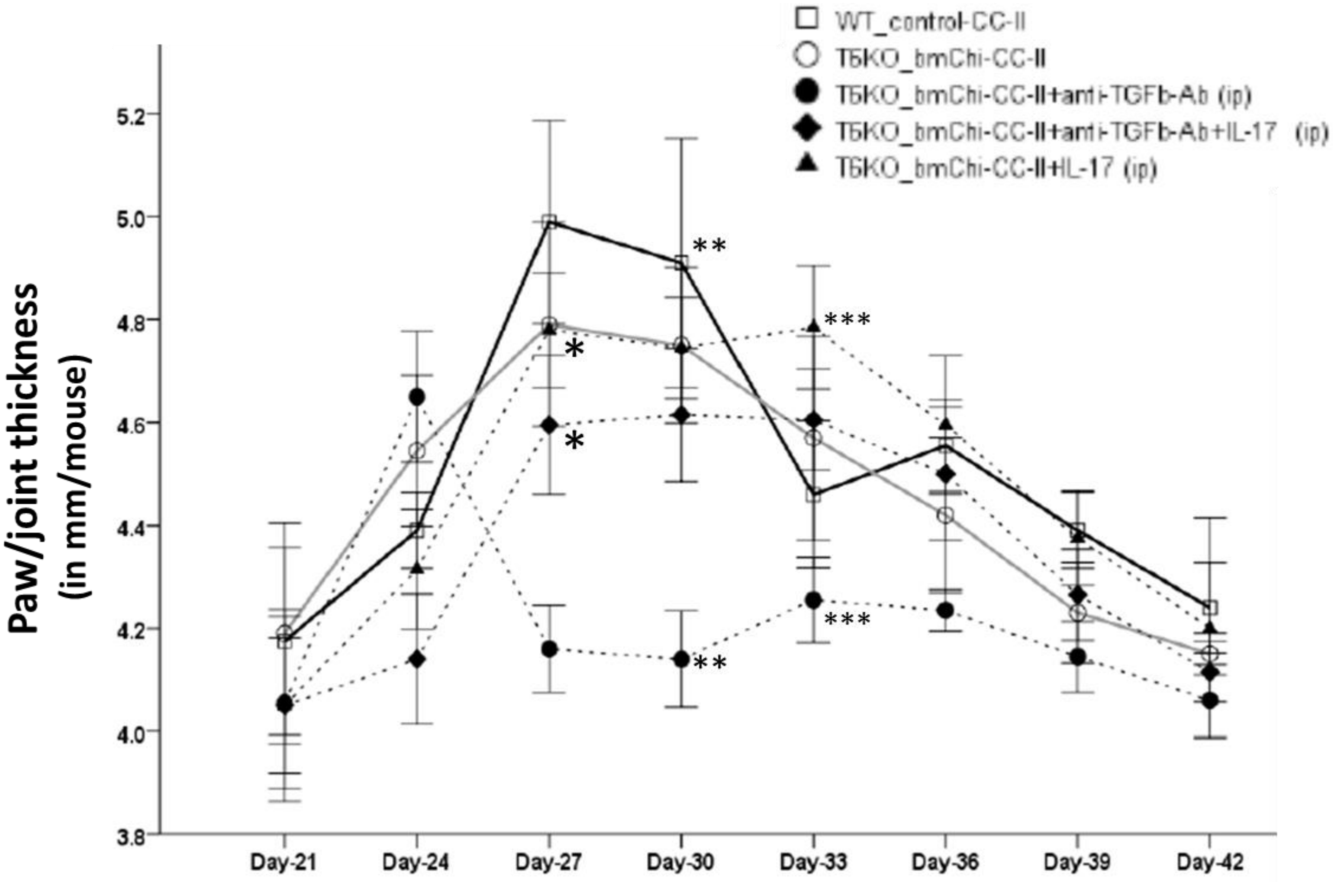

Supplemental-Fig. 1 Legend:

For measuring the joint/paws inflammation in WT- & T6KO_bmCHi chimeras, CC-II emulsified in CFA vs. IFA was employed, according to protocols described by Inglis *et al.* [**48-49**], as described in the Material & Methods section. Generally speaking, the resulting mean-scores showed their peaks occurred around day-27 to day-33 (≈wk4-5**),** with the statistically significant differences detected (***,**,***:** ***p*-values <0.0001),** among T6KO_bmChi-CC-II **(** ○ **)**, T6KO_bmChi-CC-II+anti-TGFb-Ab+IL-17-ip (♦) and T6KO_bmChi-CC-II+IL-17-ip (▲) chimeras, and specifically compared to the same date(s) from those of WT_bmChi-CC-II [as (+)-control:□**]** and T6KO_bmChi-CC-II+anti-TGFβ-Ab chimeras(●), which yielded the background levels [the paired t-test; **where ****p***<0.0092 (ranged 0.0000~0.0092); **p<0.0237 (ranged 0.0000~0.0237); ***p<0.0205 (ranged 0.0000~0.0205)** as depicted]. Additional significant differences detected among the other group comparisons are omitted in the above diagram, as Supplementary-Fig. 1. Note: Other control groups (i.e., PBS injected to WT mice transferred with WT-BM [WT_bmChi-PBS] & PBS injected to WT mice transferred with TRAF6 KO-BM [T6KO_bmChi-PBS] were prior-reported [47]; thus, omitted herein.
